# Emerging Respiratory Virus Threats from Influenza D and Canine Coronavirus HuPn-2018 

**DOI:** 10.3201/eid3201.251764

**Published:** 2026-01

**Authors:** Gregory C. Gray, Anastasia N. Vlasova, John A. Lednicky, Thang Nguyen-Tien, Ismaila Shittu, Feng Li

**Affiliations:** University of Texas, Galveston, Texas, USA (G.C. Gray, T. Nguyen-Tien, I. Shittu); The Ohio State University, Wooster, Ohio, USA (A.N. Vlasova); University of Florida, Gainesville, Florida, USA (J.A. Lednicky); University of Kentucky, Lexington, Kentucky, USA (F. Li)

**Keywords:** influenza D, viruses, canine coronavirus, CCoV, HuPn-2018, respiratory viruses, Orthomyxoviridae, Coronaviridae, emerging pathogens, zoonoses, respiratory infections, United States

## Abstract

In 2009 and again in 2019, public health warnings were confirmed by the emergence, rapid widespread transmission, and lethality of novel influenza and coronaviruses. The world continues to suffer disease from these respiratory viruses. Two newly recognized emergent respiratory viruses, influenza D and canine coronavirus HuPn-2018, have been shown to have considerable potential for causing future human epidemics, but diagnostics and surveillance for the viruses are lacking. We reviewed data regarding influenza D virus and coronavirus canine coronavirus HuPn-2018. Those data strongly indicate that these viruses are major newly recognized threats. However, little is being done to respond to or prevent disease associated with these viruses, warranting the question of whether we will learn from previous pandemics.

Although science has developed effective countermeasures for most bacterial and vectorborne emerging pathogens, novel respiratory viruses continue to cause largescale human epidemics. Particularly problematic are pathogens that are of zoonotic origin. Viruses causing epidemics seem especially common among the Orthomyxoviridae and Coronaviridae viral families (*1*–*6*; https://www.who.int/emergencies/disease-outbreak-news/item/2025-DON560; https://data.who.int/dashboards/covid19/deaths?n=o) (Table). Those epidemics have routinely caught medical professionals off-guard and caused largescale disease and death. Two recently discovered viruses, influenza D and canine coronavirus HuPn-2018 (CCoV-HuPn-2018), seem especially worthy of closer public health attention.

**Table:** Recent epidemics or pandemics from respiratory viruses thought to be zoonotic*

Period	Virus	Virus family	Deaths	Scope
1918–1920	Influenza A(H1N1)	Orthomyxoviridae	20–100 million (*1*)	Worldwide
1957–1958	Influenza A(H2N2)	Orthomyxoviridae	1–4 million (*2*)	Worldwide
1968–1969	Influenza A(H3N2)	Orthomyxoviridae	1–4 million (*3*)	Worldwide
2002–2004	SARS-CoV-1	Coronaviridae	774 (*4*)	29 countries (*5*)
2009–2010	Influenza A(H1N1)	Orthomyxoviridae	151,700–575,400 (*6*)	Worldwide
2012–present	MERS-CoV	Coronaviridae	2,627†	27 countries†
2019–present	SARS-CoV-2	Coronaviridae	7.1 million‡	Worldwide

*MERS-CoV, Middle East respiratory syndrome coronavirus; SARS-CoV, severe acute respiratory syndrome coronavirus. †Per World Health Organization (https://www.who.int/emergencies/disease-outbreak-news/item/2025-DON560). ‡Per World Health Organization as of October 2025 (https://data.who.int/dashboards/covid19/deaths?n=o).

## Influenza D Virus

**Figure 1:**
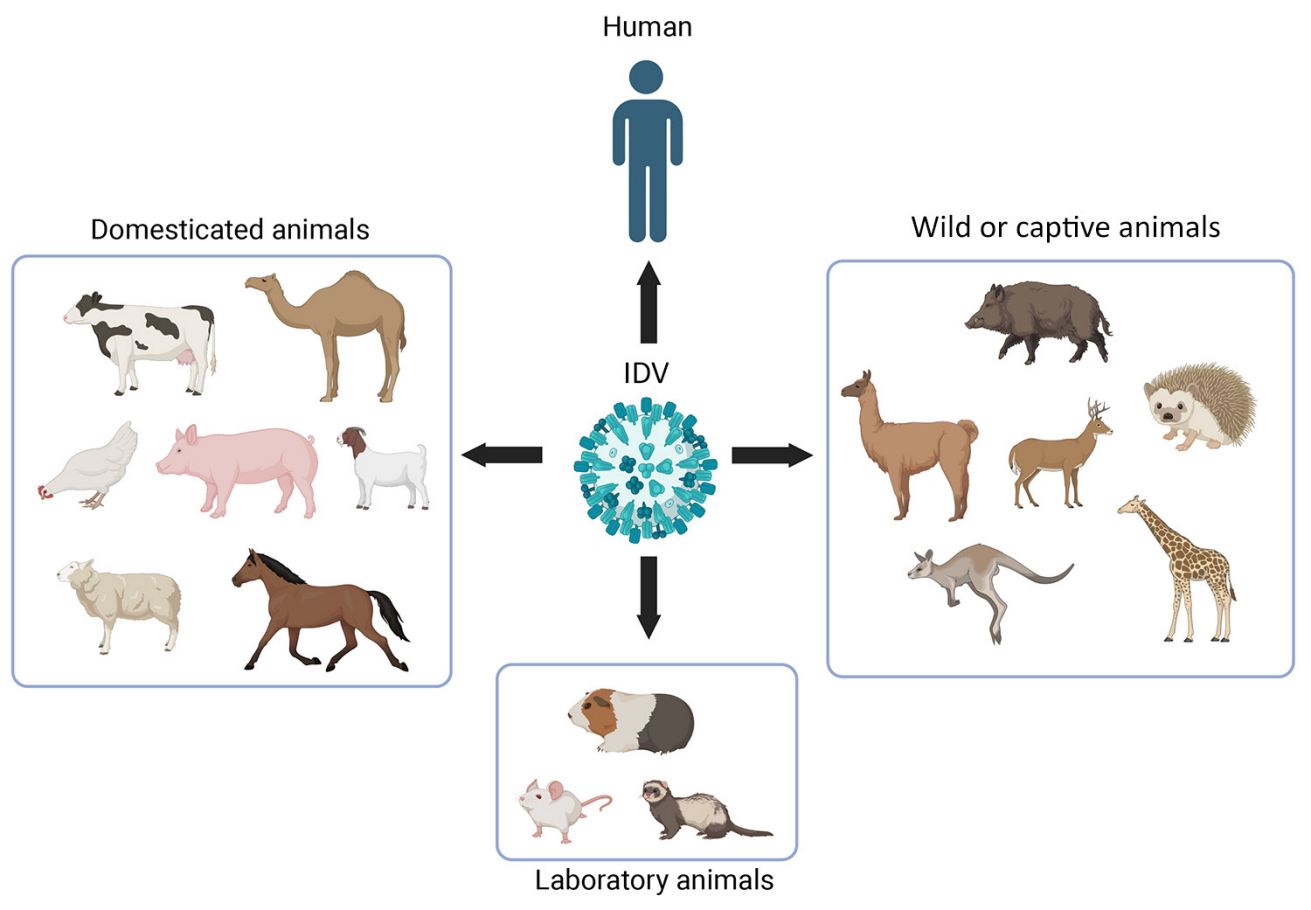
Schematic illustration of the host range of IDV. Natural infections have been confirmed through the detection of virus genomic RNA, virus isolation, or the presence of IDV antibodies in domestic animals, wild animals, captive animals, and humans (*9*–*13*). Clinically apparent infections have been observed in laboratory animals, including ferrets, guinea pigs, and mice, that have been exposed to IDV in laboratory experiments (*14*). Figure created in BioRender (https://biorender.com/uxr6kea). IDV, influenza D virus.

First detected and characterized in pigs with signs of respiratory illness in 2011, much has been learned about influenza D virus (IDV) since its first recognition (*7*,*8*). Like influenza A, B, and C viruses, IDVs are enveloped RNA viruses having segmented genomes that can change through reassortment, recombination, and mutation. IDVs belong to the genus *Deltainfluenzavirus* of the virus family Orthomyxoviridae. They share ≈50% amino acid identity with influenza C viruses (ICVs) across their genomes, but IDVs are much more prevalent in animal species. Initially thought to be enzootic in pigs and cattle, IDVs have now been detected in many livestock and wildlife species, including camels, deer, giraffes, kangaroos, llamas, wallabies, and wildebeests (*9*–*14*) (Figure 1). We have recently found evidence for IDV infections in poultry (*12*). A growing list of susceptible hosts for this new virus seems to be similar to those observed in the infection ecology of highly pathogenic avian influenza A(H5N1) viruses.

Like ICVs, a ubiquitous pathogen that causes minor influenza in humans, IDVs rely upon a hemagglutinin-esterase-fusion glycoprotein for cell binding and entry. Multiple recognized IDV strains or clades are chiefly classified by their hemagglutinin-esterase-fusion sequence circulating in animals. The genes of different IDV clades can reassort when they infect the same cell, and this mixing of genetic material leads to the generation of novel progeny viruses. Although science has had much more time to describe the ecology of ICVs, which were discovered in 1947, IDVs may reassort and recombine with other IDVs, suggesting that they are rapidly evolving (*15*). Although IDV prevalence is often high in cattle (G.C. Gray, unpub. data, 2025 Dec 11) compared with ICVs (*16*), IDVs may have more opportunity to gain characteristics that would threaten humans.

Although not all pig or cattle herds worldwide are affected by IDVs (*17*–*19*), many animal species clearly have infections periodically. The largest animal reservoir seems to be cattle, despite the first IDV being isolated from a diseased pig. The full spectrum of IDV illness in cattle is not known, but IDV is now recognized to contribute to one of the largest disease problems in cattle, bovine respiratory disease complex, which is estimated to cost the US cattle industry >$1 billion annually (*20*). As a measure of IDV endemicity, in our recent studies of 12 beef and dairy cattle farms in the United States and Mexico, we have detected (and often isolated) IDV >50 times among >500 nasal swab specimens obtained from sick or healthy cattle (G.C. Gray, unpub. data, 2025 Dec 11).

Although no viable (i.e., infectious) IDV has thus far been isolated from humans, mounting evidence indicates that the virus is zoonotic. Several human studies suggest that IDV causes subclinical infections in humans, especially among persons with occupational exposure to animals. In 2016, we reported a seroepidemiologic study of cattle workers in Florida, USA, where we found that >97% of cattle workers had neutralizing antibody to IDV, compared with 18% among a non–cattle exposed control population (*13*). In 2023, we reported a study of dairy workers in Colorado, where we found that 67% of 31 workers had molecular evidence of IDV in their nasal washes during a 5-day period (*21*).

Recently, a scientific team in China has reported compelling animal model, aerosol, and seroepidemiologic data that provide even stronger evidence that IDV is causing subclinical infections in humans (*14*). The team found that 73% of 612 study participants (97% among those with respiratory symptoms) in northeast China had serologic evidence of infection (*14*). They documented viral transmission in the air between ferrets, replication in primary human epithelial cells, infection in mice and dog models, and concluded that IDV has acquired the capacity for human-to-human transmission and that IDV strains already pose a potential panzootic threat (*14*). Of note, that study provides the first serologic evidence of widespread IDV in a general human population. The IDV strain in China that was used in the study, D/HY11, was isolated in 2023 from cattle and seems to be more efficient for airborne transmission in ferrets than strains used in previous studies. The observed increase in aerosol transmission is probably associated with mutations identified in this new strain, especially in the polymerase P3 gene, but more studies are needed to understand the underlying mechanisms behind these findings. Nevertheless, IDV replication and transmission in ferrets, 1 surrogate model for humans in influenza studies, and efficient replication in human primary airway epithelial cells observed in that work and another study (*22*) provide a theoretical framework that the virus will adapt and evolve for effective growth and human-to-human spread.

## CCoV-HuPn-2018

**Figure 2:**
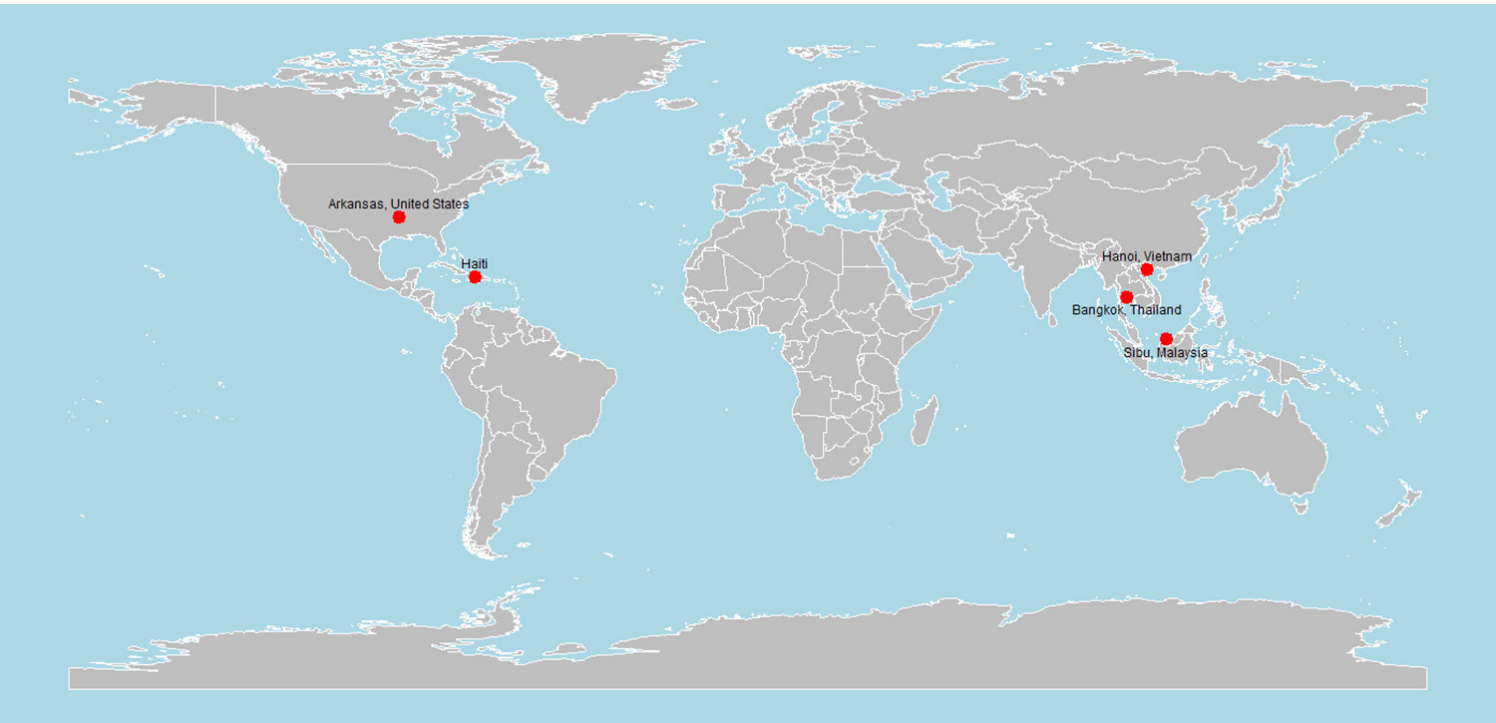
Locations of canine coronavirus HuPn-2018 or similar virus detections among humans with respiratory illness (*23*–*27*). Figure created in R version 4.4.1 (https://cran.rstudio.com).

In 2021, we first reported the cell culture isolation and characterization of a novel canine–feline recombinant alphacoronavirus, CCoV-HuPn-2018, from a nasopharyngeal swab sample from a child hospitalized with pneumonia in Sarawak State, Malaysia (*23*). The virus shared ≈97% nucleotide identity in most structural genes with canine coronavirus II, but its spike gene contained segments from feline coronavirus and transmissible gastroenteritis virus, which are specific for the canine coronavirus IIb subtype, suggesting a complex recombinant origin. A subsequent virus culture and characterization in urine specimens from persons visiting Haiti (*24*) indicated a 99.4% identity, confirming the circulation of CCoV-HuPn-2018 in different geographic regions. In addition, similar animal alphacoronaviruses have been detected among humans with respiratory illness living in Bangkok, Thailand (*25*), and in the US state of Arkansas (*26*). Recently, we detected CCoV-HuPN-2018 among 18 of 200 pneumonia patients hospitalized in the area of Hanoi, Vietnam, suggesting that this virus may have a wide geographic distribution and variable (and possibly increasing) prevalence (*27*) (Figure 2**)**. The virus is entirely missed by common clinical diagnostics tests for the detection of respiratory viruses.

Although these findings do not yet prove that CCoV-HuPn-2018 is a frequent, worldwide cause of severe respiratory disease, they suggest that CCoV-HuPn-2018 (or very similar viruses) merit our closer study. Recent studies of the spike protein of CCoV-HuPN-2018 have shed light on its interaction with the aminopeptidase N from canines, felines, and porcines, but not humans, as functional receptors for cell entry (*28*–*30*). Nevertheless, CCoV-HuPn-2018 spike protein pseudotyped virus infects multiple human cancer cell lines in a human aminopeptidase N–independent manner. Earlier clades of CCoV-HuPn-2018 might have not yet evolved to be an efficient human pathogen, but they may be evolving now, as evidenced by the increased number of patients affected by the virus in the study by our surveillance team in Vietnam (*27*).

## Other Viruses

Of course, in addition to IDV and CCoV-HuPn-2018, public health professionals should seek to detect other animal respiratory viruses as they spill over to infect humans. When possible, such surveillance should be strategically focused at the human–animal nexus where we recognize the risk is high (*31*). For instance, the risk for novel swine viruses spilling over from swine to infect swine workers is exceedingly high compared with the similar risk for avian influenza viruses spilling over from poultry to infect poultry workers (*32*–*37*). Similarly, we are aware of the zoonotic threat of other animal coronaviruses infecting persons directly or indirectly exposed to their animal hosts (*38*,*39*). In addition, we posit that evidence of animal adenoviruses spilling over to infect humans is mounting (*40*). In recent years, molecular evidence has shown that a vampire bat–like adenovirus in Malaysia (*41*) and a bovine adenovirus in Pakistan (J.R.E. Ansari et al., unpub. data, https://doi.org/10.21203/rs.3.rs-5811360/v1) were associated with human respiratory disease. Because of those and other observations, we argue that periodic surveillance with targeted and panspecies diagnostics would be prudent when addressing emerging respiratory virus threats for viruses in 6 viral families (Adenoviridae, Coronaviridae, Orthomyxoviridae, Paramyxoviridae, Picornaviridae, and Pneumoviridae) (*42*). Conducting such surveillance in concert with occasional agnostic next-generation sequencing of specimens associated with unusual illnesses can help us better prepare for future pandemic threats at more sustainable costs than previous strategies that sought to detect novel pathogens in many wildlife hosts (*43*).

## Conclusions

The novelist Stephen King adapted a classical English language quip to illustrate our tendency to ignore our previous mistakes: “Fool me once, shame on you. Fool me twice, shame on me. Fool me three times, shame on both of us” (*44*). As we prepare for the next pandemic, we would be wise to heed his advice.

Currently, we know of no human or veterinary laboratory-approved molecular or serologic assays for IDV or CCoV-HuPN-2018. Hence, our knowledge about the viruses’ epidemiology and clinical manifestations are limited to a modest number of research studies. Even so, the limited data regarding these novel, newly detected viruses indicate that that they are a major threat to public health. If we wish to avoid being fooled again by a novel virus suddenly gaining efficient human-to-human transmissibility and causing large human epidemics, we would be wise to develop better surveillance systems and new countermeasures for these and similar viruses. Potential actions include the development of commercial real-time reverse transcription PCR diagnostic tests specifically targeting IDV and CCoV-HuPN-2018 viruses. Conducting periodic assessments for novel respiratory viruses could detect what might be cryptic causes of hospitalizations or animal epizootics in geographic areas recognized to be sites of increased presence of emerging pathogens (*42*,*45*). As we have shown, this surveillance can easily be performed with panspecies and next-generation sequencing diagnostics (*42*,*46*,*47*). Clinicians should consider IDV and CCoV-HuPN-2018 in their workup of pneumonia patients when a primary battery of diagnostic tests fail to identify a pathogen. Scientists should begin evaluating antiviral drugs as effective therapy for the treatment of IDV and CCoV-HuPN-2018 infections. If further epidemiologic study indicates that the investment is warranted, human vaccine development should be considered for both IDV and CCoV-HuPN-2018. Furthermore, with respect to detecting new respiratory virus threats, when specific novel animal spillover risks are identified, they may often be mitigated with targeted interventions such as those recently reviewed by Vora et al. (*48*) and Plowright et al. (*49*).
